#  Expression profiles of muscle disease-associated genes and their isoforms during differentiation of cultured human skeletal muscle cells

**DOI:** 10.1186/1471-2474-13-262

**Published:** 2012-12-29

**Authors:** Saba Abdul-Hussein, Peter F M van der Ven, Homa Tajsharghi

**Affiliations:** 1 Department of Pathology, University of Gothenburg, Sahlgrenska University Hospital, Gothenburg, SE, 413 45, Sweden; 2 Department of Molecular Cell Biology, Institute for Cell Biology, University of Bonn, Bonn, 53121, Germany; 3 Department of Clinical and Medical Genetics, University of Gothenburg, Sahlgrenska University Hospital, Gothenburg, SE, 413 45, Sweden

**Keywords:** Myogenesis, Sarcomere, Myoblast, Skeletal muscle

## Abstract

**Background:**

The formation of contractile myofibrils requires the stepwise onset of expression of muscle specific proteins. It is likely that elucidation of the expression patterns of muscle-specific sarcomeric proteins is important to understand muscle disorders originating from defects in contractile sarcomeric proteins.

**Methods:**

We investigated the expression profile of a panel of sarcomeric components with a focus on proteins associated with a group of congenital disorders. The analyses were performed in cultured human skeletal muscle cells during myoblast proliferation and myotube development.

**Results:**

Our culture technique resulted in the development of striated myotubes and the expression of adult isoforms of the sarcomeric proteins, such as fast TnI, fast TnT, adult fast and slow MyHC isoforms and predominantly skeletal muscle rather than cardiac actin. Many proteins involved in muscle diseases, such as beta tropomyosin, slow TnI, slow MyBPC and cardiac TnI were readily detected in the initial stages of muscle cell differentiation, suggesting the possibility of an early role for these proteins as constituent of the developing contractile apparatus during myofibrillogenesis. This suggests that in disease conditions the mechanisms of pathogenesis for each of the mutated sarcomeric proteins might be reflected by altered expression patterns, and disturbed assembly of cytoskeletal, myofibrillar structures and muscle development.

**Conclusions:**

In conclusion, we here confirm that cell cultures of human skeletal muscle are an appropriate tool to study developmental stages of myofibrillogenesis. The expression of several disease-associated proteins indicates that they might be a useful model system for studying the pathogenesis of muscle diseases caused by defects in specific sarcomeric constituents.

## Background

The formation of skeletal muscle cells takes place during embryogenesis, postnatal growth and repair of postnatal skeletal muscle. Postnatal growth of muscle and regeneration of adult skeletal muscle tissue following injury is accomplished by activation of quiescent satellite cells that subsequently proliferate, differentiate and fuse with each other or pre-existing myofibers to regenerate myofibers thereby regenerating the muscle tissue [1]. For these characteristics of self-renewal, cultured satellite cells can provide an invaluable insight into the basic mechanisms of sarcomere expression profile, assembly and myofibrillogenesis. Although previous studies have revealed some distinct genetic requirements for embryonic, fetal, postnatal and adult regenerative myogenesis, the basic mechanisms are likely to be similar [2,3].

Striated muscle formation is a fundamentally conserved process and requires the highly regulated and ordered expression of sarcomeric proteins and their assembly into the sarcomeric contractile unit [4]. The principle components of striated muscle sarcomeres include parallel arrays of actin-containing thin filaments, which overlap with myosin-containing thick filaments. Striated muscle function depends on the precise alignment of actin and myosin filaments, which is achieved by accessory proteins that link the different components and hold them in register with each other [5]. In the sarcomere many proteins cooperate to convert the molecular interactions of actin and myosin efficiently into mechanical force and movement [4].

A common property of many of the sarcomeric components of striated muscle is that they often exist as families of similar isoforms. These isoforms are developmentally regulated and differentially expressed in a tissue-specific manner in cardiac, slow and fast muscle [6,7]. The generation of distinct protein isoforms within a family can result predominantly by two mechanisms: the differential expression of multigene families or the production of multiple protein variants from a single gene via alternative splicing. Muscle proteins, which exist as various isoforms in developing and mature skeletal and cardiac muscles are functionally unique and exhibit distinct contractile and physiological properties [8-10].

Although several studies have described that during myofibrillogenesis, the expression of proteins occurs in an ordered sequence [4], the sequential onset of the individual isoforms within a family of sarcomeric proteins is poorly characterized. Elucidation of the expression patterns of sarcomeric proteins and their distinct isoforms during myogenesis, is important to better understand their function, formation and assembly into sarcomeric structure in normal muscle cells. These data will allow a comparison with muscle cells obtained from patients suffering from one of the increasing number of diseases originating from genetic defects in these proteins. Congenital myopathies, a heterogeneous group of muscle disorders defined by distinctive morphologic abnormalities in skeletal muscle fibers, are often caused by structural defects in sarcomeric or cytoskeletal proteins [11]. In addition, distal arthrogryposis (DA) syndromes, a heterogeneous group of disorders with congenital contractures mainly of hands and feet, have recently been associated with mutations in sarcomeric components including ß-tropomyosin (*TPM2*) [12,13], fast troponin T (*TNNT3*) [14], fast troponin I (*TNNI2*) [12,15], embryonic myosin heavy chain (*MYH3*) [16,17], prenatal myosin heavy chain (*MYH8*) [18] and slow myosin binding protein C (*MYBPC1*) [19].

In the present study, we characterize the expression profile of a panel of muscle-specific sarcomeric components that are associated with congenital diseases by combining molecular and morphological techniques on cultured human adult regenerative myoblasts and differentiated myotubes.

## Methods

### Muscle cell Cultures

Five standardized batches of human myoblasts were provided by MYOSIX through a collaborative program with Association Francaise contre les Myopathies (AFM). Skeletal muscle cells from donors with no clinical signs of muscle disease were enzymatically isolated and cultured as previously described [20,21]. Isolated satellite cells, frozen in liquid nitrogen in their second passage, were quickly thawed and plated on chamber slides (Lab-Tek™ II - CC2™, Nalge Nunc International, Naperville, USA) using Dulbecco’s modified Eagle’s medium (DMEM) (Biochrom AG, Berlin, Germany), supplemented with 20% fetal bovine serum (Invitrogen, GIBCO, Auckland, New Zealand), 100x GlutaMAX (Invitrogen, GIBCO, Paisley, UK), 10 mg/ml penicillin/streptomycin (Biochrom AG, Berlin, Germany), 10 μm./ml insulin (Biochrom AG, Berlin, Germany) and 10 ng/ml fibroblast growth factor (BD Biosciences, Bedford, MA, USA). The cells were incubated at 37°C in a humidified 5% CO_2_ atmosphere.

To induce differentiation, cells were replenished at 85%-90% confluence with DMEM medium, supplemented with 5% horse serum (Invitrogen, GIBCO, Auckland, New Zealand) and further incubated in this medium for 6 days. The medium was changed three times a week.

### RNA isolation, cDNA synthesis, polymerase chain reaction (PCR) and sequence analysis

Total RNA was isolated from proliferating myoblasts and differentiated cell cultures and treated with DNase I using the RNeasy Plus Mini Kit (Qiagen, Hilden, Germany) according to the manufacturer’s instructions. The total RNA concentration was determined by A_260_ and A_280_ (A_260_/A_280_=1.7-2.0) measurements using a NanoDrop ND 1000 Spectrophotometer (NanoDrop Technologies, Wilmington, USA). Synthesis of first-strand complementary DNA (cDNA) was performed from 400 ng total RNA using the iScript cDNA Synthesis kit (BioRad Laboratories, 2000 Alfred Nobel Drive Hercules, CA), according to the manufacturer’s instructions.

The expression of transcripts of a panel of genes encoding skeletal muscle sarcomeric components desmin (*DES*), titin (*TTN*), MyHC isoforms (*MYH1, MYH2, MYH3, MYH4, MYH7* and *MYH8*), α-skeletal actin (*ACTA1*) and α-cardiac actin (*ACTC1*), tropomyosin isoforms (*TPM1, TPM2* and *TPM3*), troponin T isoforms (*TNNT1, TNNT2* and *TNNT3*), troponin I isoforms (*TNNI1, TNNI2* and *TNNI3*), skeletal muscle myosin-binding protein C isoforms (*MYBPC1, MYBPC2*) and cardiac myosin-binding protein C (*MYBPC3*) was analyzed by reverse transcriptase polymerase chain reaction (PCR). In addition, the expression of transcripts of myogenic regulatory factors (MRFs) myogenic factor 5 (*MYF5*), myogenic differentiation 1 (*MYOD1*) and myogenin (*MYOG*) was analyzed.

The PCR analysis was performed on cDNA in a master mixture (ReddyMix PCR Master Mix, ABgene House, Blenheim Road, UK) containing 20 pmol of each primer. The PCR primers were directed to cDNA sequence flanking exons in order to exclude genomic DNA amplification, although the RNA samples were treated with DNase I. Primer sequences are available on request. PCR amplifications consisted of an initial preheating step for 5 min at 94°C, followed by a touchdown (TD)-PCR. TD-PCR analysis was performed by denaturation at 94°C for 30 sec, annealing at 65°C for 30 sec, extension at 72°C for 1 min with a 1°C temperature decrement per cycle during the first 10 cycles. The subsequent cycles (40 cycles) each consisted of 94°C for 30 sec, 55°C for 30 sec and 72°C for 1 min. The same PCR amplification reaction was performed for all genes, except for the amplification of myosin heavy chain isoforms that consisted of an initial preheating step for 3 min at 94°C, followed by denaturation at 94°C for 1 min, annealing at 57°C for 1 min and extension at 72°C for 1 min for 35 cycles. Samples were run in triplicate. The nucleotide sequence determination was carried out by cycle sequencing using an ABI 3730xl DNA sequencer (GATC Biotech AG, Konstanz, Germany).

### Immunocytochemistry and immunofluorescence

Immunocytochemical analysis was performed on proliferating myoblasts and myotubes after 6 days of differentiation, grown on chamber slides using LSAB kit (DAKO, Glostrup, Denmark) according to manufacturer’s instructions. The cells were incubated with primary antibodies (Table 1) followed by incubation with EnVision™Flex/HRP. The immunoreactivity was visualized by the indirect peroxidase-antiperoxidase complex method (DAKO, Glostrup, Denmark) using diaminobenzidine as a chromogen. Nuclei were counterstained with hematoxylin (Novocastra, Leica Microsystems Newcastle Ltd). Immunoreactivity was analyzed by light microscopy using the Zeiss Axio Observer microscope (Carl Zeiss AG, Germany) equipped with 10×, 20× and 40× objectives.

**Table 1:** Antibodies used for the immunocytochemical and immunofluorescence analyses

**PRODUCT NAME**	**PRODUCT CODE**	**ANTIGEN**	**DILUTION IMMUNOCYTO CHEMISTRY/IMMUNOFLUORESCENCE**	**DILUTION SECONDARY ANTIBODY**	**COMPANY**
Mouse monoclonal to desmin	M0760	Desmin	1:100	1:1000 Anti-mouse Dylight 549	DakoCytomation
Mouse monoclonal [F5D] to myogenin	Ab1835	Myogenin	1:50		Abcam plc
Mouse monoclonal to troponin T, fast	NCL-TROPT	TNNT	1:20		NovoCastra™Lyophilized
Mouse monoclonal to titin	3010-S	Titin	1:50		BioCytex
Mouse monoclonal to sarcomeric actin	M0874	Alpha-skeletal actin	1:10	1:1000 Anti-mouse Dylight 549	DakoCytomation
Mouse monoclonal cardiac actin	M622709	Alpha-cardiac actin	1:20	1:1000 Anti-mouse Dylight 549	Nordic Biolabs Bioreagents
Mouse monoclonal to myosin heavy chain, (developmental)	NCL-MHCd	MyHC-embryonic	1:10	1:1000 Anti-mouse Dylight 549	NovoCastra™Lyophilized
Mouse monoclonal to myosin heavy chain, (neonatal)	NCL-MHCn	MyHC-neonatal	1:10	1:1000 Anti-mouse Dylight 549	NovoCastra™Lyophilized
Mouse monoclonal to myosin heavy chain, (slow)	NCL-MHCs	MyHC-slow	1:250		NovoCastra™Lyophilized
Mouse monoclonal to myosin heavy chain, (fast)	NCL-MHCf	MyHC-fast	1:120		NovoCastra™Lyophilized
Mouse monoclonal to myosin heavy chain, (fast IIa and slow)	N2.261	MyHC-fast IIa+slow	1:120	1:1000 Anti-mouse Dylight 549	Santa Cruz Biotechnology
Mouse monoclonal to sarcomeric tropomyosin	T9283	TPM	1:100		Sigma-Aldrich
Rabbit polyclonal to beta tropomyosin	ARP48224T100	TPM2	1:120		Aviva Systems Biology
Rabbit polyclonal to myosin binding protein C, (slow)	HPA021004	MYBPC1-slow	1:50		Sigma-Aldrich
Rabbit polyclonal to myosin binding protein C, (fast)	SAB2101539	MYBPC2-fast	1:100		Sigma-Aldrich
Mouse monoclonal [12F10] to skeletal muscle troponin I, (slow)	Ab8293	TNNI-slow	1:500		Abcam plc
Mouse monoclonal [2F12A11] to skeletal muscle troponin I, (fast)	Ab119943	TNNI- fast	1:200		Abcam plc
Mouse monoclonal [284(19C7)] to cardiac troponin I	Ab19615	TNNI- cardiac	1:500		Abcam plc
Mouse monoclonal T12 to Z-disk titin		Z-disk	1:20	Polyclonal Anti-Mouse Immunoglobulins/FITC 1:1000	Fürst et al., 1988
Mouse monoclonal T3 to A/I junction titin		A/I junction	1:5	Polyclonal Anti-Mouse Immunoglobulins/FITC 1:1000	Fürst et al., 1988
Mouse monoclonal T30 to A-band titin		A-band	1:5	Polyclonal Anti-Mouse Immunoglobulins/FITC 1:1000	Fürst et al., 1989
Mouse monoclonal T51 to M-band titin		M-band	1:5	Polyclonal Anti-Mouse Immunoglobulins/FITC 1:1000	Obermann et al., 1996

For immunofluorescence assays, slides were washed three times in 0.01 M phosphate-buffered saline (PBS) and fixed in 4% formaldehyde (methanol-free 16% formaldehyde solution, Thermo scientific) for 10 min. Free aldehyde groups were blocked with 50 mM NH_4_Cl for 10 min and cells were permeabilized in 0,01 M PBS containing 0,1% Triton X-100 for 4 min. Cells were incubated with primary antibodies for 1 h in a humidified chamber at 37°C, followed by incubation with secondary antibodies for 1 h in the dark and three washes with 0.01 M PBS. Finally, the slides were mounted with a coverslip using Prolong® Gold antifade reagent with DAPI (Invitrogen, GIBCO, Auckland, New Zealand) to highlight cell nuclei. The slides were left overnight in the dark at room temperature before examinations.

## Results

The experiments were conducted in triplicate with similar results.

### RNA expression and sequence analysis

**Figure 1:**
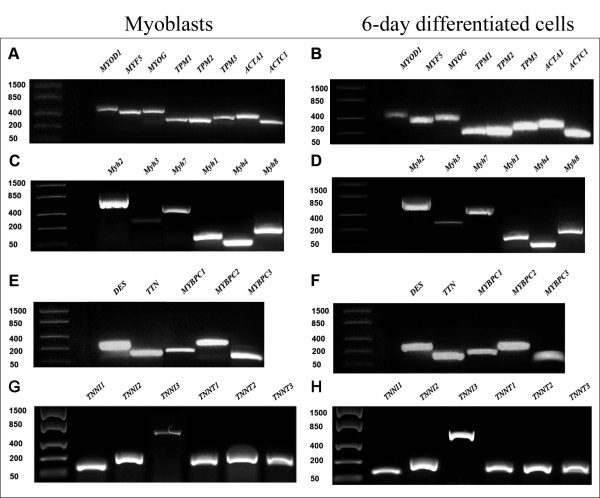
**RT-PCR analysis of myogenic regulatory factors (MRFs) and a panel of striated muscle sarcomeric genes in myoblasts and cells differentiated for 6 days.** RNA isolated from proliferating mononucleated myoblasts (**A**, **C**, **E** and **G**) and cultures after 6 days of differentiation (**B**, **D**, **F** and **H**) was analyzed by RT-PCR and the products were separated on agarose gels. The expression of MRF genes (*MYOD1*, *MYF5* and *MYOG*), TM isoforms, *ACTA1, ACTC1*, different MyHC isoforms, *DES, TTN* and MyBPC isoforms, and troponin I and T isoforms was readily detectable in myoblasts and differentiated cells.

The appearance of the myogenic phenotype in the investigated cell cultures was verified by analyzing the expression of the genes encoding a family of transcription factors known as MRFs including *MYOD1, MYF5* and *MYOG* that are responsible for controlling muscle-specific gene expression. Expression of all MRFs was readily detectable in both proliferating mononucleated myoblasts and cells after 6 days of differentiation (D6) (Figure 1A and B).

The expression of tropomyosin isoforms *TPM1, TPM2, TPM3,* α-skeletal and α-cardiac actin (*ACTA1* and *ACTC1*), MyHC isoforms (*MYH1, MYH2, MYH3, MYH4, MYH7*, *MYH8*), *DES, TTN,* slow, fast and cardiac myosin-binding protein C isoforms (*MYBPC1, MYBPC2, MYBPC3*), troponin I isoforms (*TNNI1, TNNI2*, *TNNI3*)*,* and troponin T isoforms (*TNNT1, TNNT2, TNNT3*) was detected in both myoblasts and D6 cells (Figure 1A-H).

In order to confirm and verify the expression of each specific transcript in both myoblasts and differentiated cells, a sequence analysis of the amplicons was performed. Using the NCBI BLAST website http://www.ncbi.nlm.nih.gov/BLAST/, the 100% identity to the reference sequences were confirmed for the MRF transcripts (*MYOD1*, *MYF5 and MYOG*), as well as the muscle specific alternative splicing variant of the investigated sarcomeric transcripts (Additional files 1, 2, 3, Figure S1–S3).

### Immunocytochemistry and immunofluorescence

**Figure 2:**
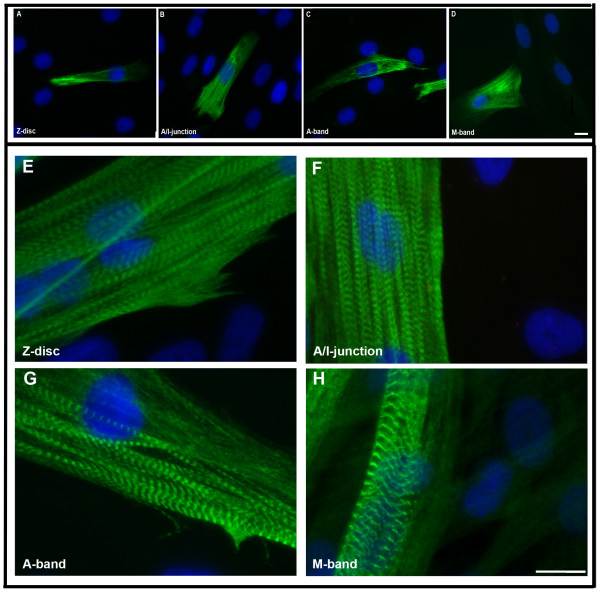
**Immunofluorescence micrographs of stained myoblasts and 5-day myotube cultures.** Staining with monoclonal antibodies recognizing titin epitopes in the Z-disk (**A** and **E**), A/I junction (**B** and **F**), A-band (**C** and **G**) and M-band (**D** and **H**) reveal development of mature cross-striated myofibrils. Nuclei were stained with DAPI (blue). The bars represent 10 μm.

Mononucleated myoblasts were differentiated into multinucleated cells to induce the development of myotubes containing cross-striated myofibrils. The mature sarcomeric cross-striated pattern of the myofibrils within differentiated cells was confirmed by immunostaining with a panel of titin antibodies: all sarcomeric structures (Z-disk, A/I-junction, A-band and M-band) were clearly detected after only 5 days of differentiation (Figure 2).

**Figure 3:**
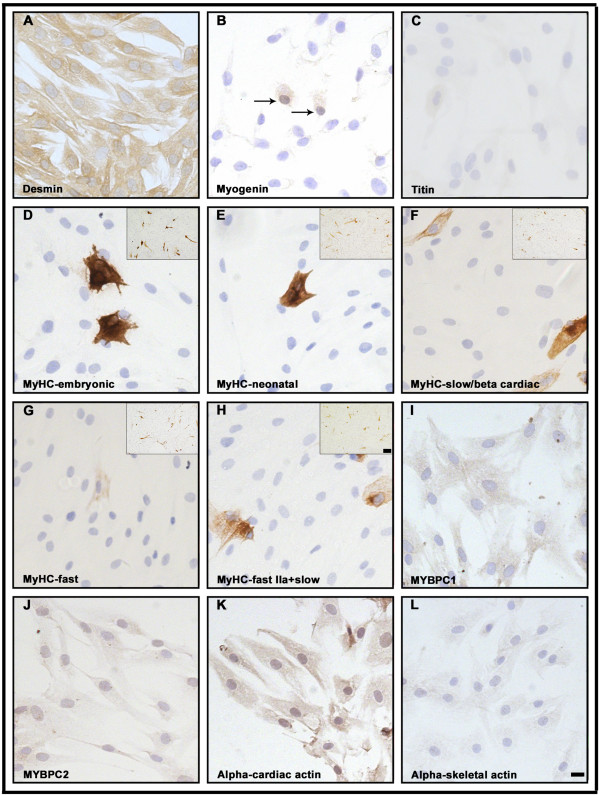
**Immunocytochemical analysis of proliferating human mononucleated myoblasts.** (**A**) The strong homogeneous staining for desmin revealed the myogenic potential of virtually all mononucleated cells. (**B**) Only a small number of the myonuclei of proliferating cells expressed myogenin (arrows). (**C**) A subset of myoblasts showed immunoreactivity with antibody against titin. (**D**-**E**-**F**-**G**-**H**) Demonstrate positive staining of a few cells with antibodies against MyHC-embryonic, MyHC-neonatal, MyHC-slow/beta cardiac, MyHC-fast and MyHC-fast IIa+slow, respectively. Insets demonstrate considerable numbers of mononucleated myoblasts expressing various MyHC isoforms, as indicated. Homogeneous staining of proliferating mononucleated cells with antibodies against slow (**I**) and fast (**J**) MyBPC respectively. Uniform and strong staining of proliferating myoblasts with antibody against alpha-cardiac actin (**K**). The cells were weakly stained with antibody against alpha-skeletal actin (**L**). The bar represents 10 μm and 100 μm for the insets.

**Figure 4:**
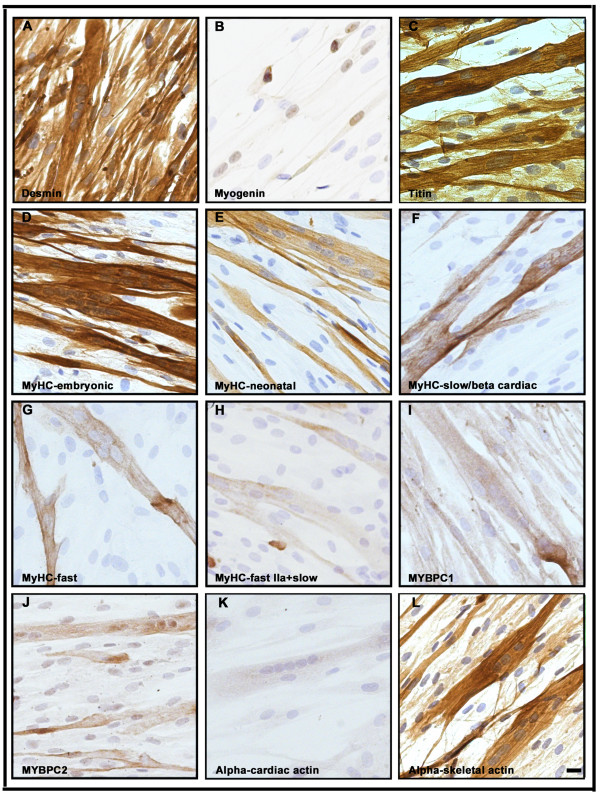
**Immunocytochemical analysis of human cells after 6 days of differentiation.** (**A**) shows strong homogeneous staining for desmin of the cells. (**B**) The differential capacity of the multinucleated cells was demonstrated by myogenin-positive staining of the majority of the nuclei. (**C**) Strong homogeneous staining of differentiated cells with an antibody against titin. (**D**) The vast majority of differentiated cells showed strong staining with the antibody against MyHC-embryonic and (**E**) many myotubes expressed neonatal MyHC. (**F-H**) expression of fast and slow MyHC was revealed in a subset of fused cells using antibodies against these isoforms. Positive staining of differentiated cells with antibodies against slow (**I**) and fast (**J**) MyBPC, respectively. The differentiated cells showed weak positive immunoreactivity with the antibody against alpha-cardiac actin (**K**) but stronger positive staining with alpha-skeletal actin (**L**). The bar represents 10 μm.

To confirm the myogenic identity of the analyzed cells they were stained with an antibody against desmin. The expression of desmin was homogeneous and the antibody strongly stained virtually all cells of both proliferating myoblasts (Figure 3A) and differentiated (D6) cultures (Figure 4A). In order to determine the stage of differentiation of the mononucleated myoblasts and the differentiated cells, an antibody against myogenin was used. Only a small number of the myonuclei of proliferating mononucleated cells were found to express myogenin (Figure 3B), whereas the majority of the nuclei were myogenin positive in the multinucleated differentiated cells confirming their more advanced differentiation level (Figure 4B).

In order to examine the sequential onset of the expression of sarcomeric proteins and their isoforms, we performed immunocytochemical analyses on mononucleated myoblasts and differentiated cells using a large panel of antibodies (Table 1). While the expression of titin was observed in a subset of myoblasts (Figure 2A-D and 3C), this protein was expressed and organized into a cross-striated pattern in differentiated cells (Figure 2E-H and 4C). The expression analysis of various MyHC isoforms was performed using five different MyHC antibodies. Although MyHC isoforms were predominantly detected in differentiated cells, about 10% of mononucleated myoblasts also expressed embryonic, neonatal, slow and fast MyHC (Figure 3D-H, insets). The vast majority of multinucleated myotubes stained strongly with the antibody against embryonic MyHC (Figure 4D-H). The expression pattern of slow and fast MyBPC was studied using two specific antibodies. While virtually all proliferating mononucleated and differentiated cells appeared to be stained with the antibody against slow MyBPC, these cells were weakly stained with the antibody against fast MyBPC (Figure 3I-J and 4I-J). In addition, no specific staining of proliferating mononucleated myoblasts or multinucleated differentiated cells with cardiac-specific MyBPC antibody was detected (data not shown).

Immunohistochemical analysis with alpha-cardiac and alpha-skeletal actin antibodies revealed uniform staining of proliferating myoblasts (Figure 3K and L). While strong expression of alpha-cardiac actin was detected in the myoblasts (Figure 3K), these cells appeared weakly stained by the alpha-skeletal actin antibody (Figure 3L). In contrast, in multinucleated differentiated cells the intensity of staining for alpha-skeletal actin appeared to be much stronger than for alpha-cardiac actin (Figure 4K and L).

**Figure 5:**
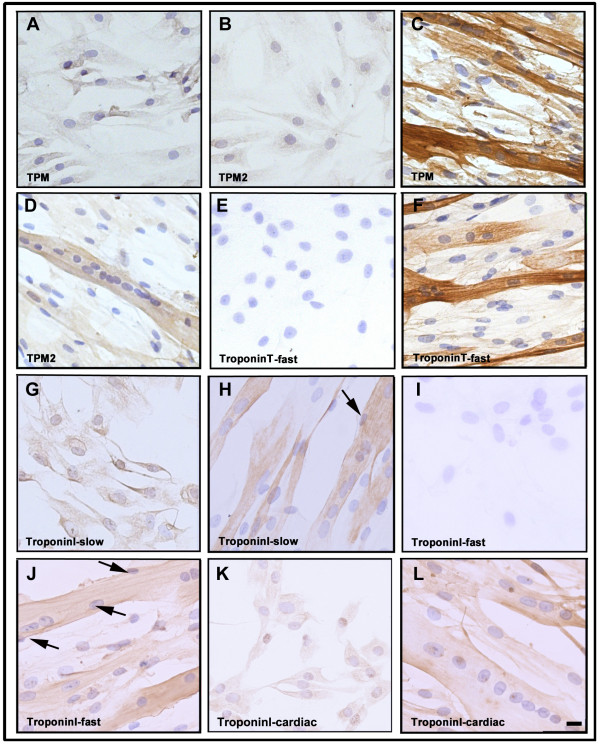
**Immunohistochemical analysis of human myoblasts and cells after 6 days of differentiation.** Homogeneous staining of cells with (**A**) antibody against three different sarcomeric TM isoforms and (**B**) a specific antibody against beta-tropomyosin, β-TM isoform. (**C**) Strong positive staining of multinucleated cells with an antibody against sarcomeric TM isoforms. (**D**) Staining of cells with specific antibody against beta-tropomyosin. (**E**) The mononucleated cells were negative for fast troponin T expression, while positive immunoreactivity was observed in (**F**) multinucleated cells, as illustrated by the anti-fast TNNT MAb staining. Homogeneous staining of (**G**) proliferating myoblasts and (**H**) multinucleated myotubes with antibody against slow isoform of troponin I. No expression of fast troponin I was detected in (**I**) proliferating myoblasts whereas positive staining was observed in (**J**) differentiated cells by an antibody against this isoform. Homogeneous immunoreactivity of both (**K**) proliferating myoblasts and (**L**) multinucleated cells with antibodies against cardiac isoform of troponin I. A subset of the multinucleated myotubes have a myofibre-like appearance with peripherally located nuclei (**H** and **J**; arrows). The bar represents 10 μm.

The expression of tropomyosin isoforms was studied in both myoblasts and differentiated cells using two different antibodies. The monoclonal anti-sarcomeric tropomyosin antibody detects all three TM isoforms (α-, β- and γ-TM isofoms), whereas the TPM2 antibody is specific for the β-TM isoform. Although both antibodies produced uniform staining of proliferating mononucleated cells (Figure 5A and B), the intensity of staining of multinucleated cells appeared to be much stronger (Figure 5C and D). While expression of the fast TnT isoform was undetectable in proliferating myoblasts, strong expression was detected in multinucleated cells (Figure 5E and F). To assess the expression of TnI isoforms, specific anti-slow and anti-fast troponin I antibodies were used. Expression of slow TnI isoforms was detected in both mononucleated and multinucleated differentiated cells (Figure 5G and H), whereas the fast TnI isoform was solely expressed in myotubes (Figure 5I and J). Notably, a subset of the multinucleated myotubes formed myofibre-like cells with peripheral nuclei (Figure 5H and J; arrows). Immunocytochemical analyses of cardiac TnI confirmed a uniform staining of proliferating mononucleated myoblasts (Figure 5K) and a vast majority of myotubes (Figure 5L). Immunofluorescence analysis confirmed these results (Additional file 4: Figure S4).

#### Supplemental information

Supplemental Appendix includes four figures.

## Discussion

Skeletal muscle myoblasts are capable of proliferation and differentiation *in vitro*, which imitates early embryonic development and muscle regeneration. In several studies mammalian or avian cultured myocytes have been used as an experimental model to analyze the temporal and spatial expression of myofibrillar proteins during myogenesis in cardiac and skeletal muscles [1,4,22-25]. These studies have lead to the understanding that during myofibrillogenesis, the initiation of the expression of the many proteins involved occurs in an ordered sequence, suggesting a specific impact of the individual proteins in the complex mechanisms associated with sarcomere formation [4]. Due to their limited availability and reduced level of differentiation, human cells were only used in few studies. A better knowledge of their expression patterns and contribution to the assembly of cytoskeletal and myofibrillar structures in human cells might provide insights into the pathomechanisms of diseases associated with mutations in genes encoding sarcomeric proteins. In the current study we have used cultured primary human skeletal muscle cells to investigate the expression patterns of a panel of sarcomeric components and their isoforms with a focus on proteins associated with a group of muscle diseases. It is of interest to understand the onset of the expression of such proteins related to the congenital feature of the disease. Our differentiated cultures were predominantly occupied by myotubes, showing a mature sarcomeric cross-striated pattern. We observed a subsequent expression and development stages of myofibril components from an initial unorganized pattern in myoblasts, into a mature cross-striated pattern with clearly distinguishable Z-disks, A/I-junctions, A-bands and M-bands. The appearance of these four well-defined sarcomeric structures, and in particular, the integration of M-band titin, which is a late stage in myofibrillogenesis [26], indicated the formation of mature sarcomeric structures *in vitro*. This indicates the high quality of our human tissue culture and confirms its usability as a model system for studying the pathogenesis of muscle diseases caused by defects in sarcomeric or cytoskeletal constituents during skeletal muscle development.

Our results identify α-cardiac actin and α-skeletal actin as the predominant actin isoform in mononucleated myoblasts and in multinucleated differentiated cells, respectively demonstrating that the expression of actin isoforms is developmentally regulated in a temporal, tissue-specific manner. This is in accordance with previous results in chicken and mouse (27–29), that revealed that α-cardiac actin is the main isoform in early skeletal muscle development [27,28]. Its expression is down-regulated in later development and α-skeletal actin becomes the predominant isoform in adult skeletal muscle fibres [29].

We also show the predominant expression of the β-TM isoform in proliferating human myoblasts and myotubes during myogenesis *in vitro*. This indicates an important role for β-TM in early stages of myofibrillogenesis. In addition, our data indicate that myoblasts and early-differentiated myotubes contain predominantly slow TnI, suggesting the importance of this isoform during development. This is further supported by the previous notion that the slow troponin I gene is the major isoform in early fetal heart in vertebrates and it is predominantly expressed during development in fast muscles with a subsequent switch to fast troponin I [30-32]. Similarly, we detected the expression of fast TnI and T solely in differentiated myotubes, indicating their impact in later stages of muscle development. Moreover, in accordance with previous results [33] the homogeneous expression of cardiac TnI in proliferating myoblasts and early myotubes suggest a role for this protein in the initial stages of myofibrillogenesis.

Association of mutations in slow skeletal muscle MyBPC isoform (*MYBPC1*) with autosomal dominant DA type 1 has recently been reported [19]. We identified this specific isoform as the major MyBPC variant at the initial phases of myofibrillogenesis in human skeletal muscle myoblasts and early myotubes, indicating that it is the main MyBPC isoform involved in early myofibril development. Accordingly, previous results indicate that slow MyBPC is first expressed in developing skeletal muscle both in mice and humans and fast MyBPC is detected at later developmental stages [3]. Also in accordance with a previous study, which indicated that cardiac MyBPC is not expressed in human skeletal muscles, not even during development [3], the expression of this isoform was not detected in our cultures. This indicates that the cardiac MyBPC appears not to be essential for human skeletal muscle development. However the cardiac MyBPC transcript was clearly detected in both, proliferating mononucleated myoblasts and myotubes, indicating the ectopic expression of this gene, as previously suggested [34]. In addition, we observed the expression of muscle-specific sarcomeric transcripts in proliferating mononucleated myoblasts, which either suggests the existence of myoblasts that had begun to express these components prior to fusion, or an expression of muscle-specific proteins in mononucleated myoblasts that differentiate prematurely. We found early expression of various MyHC protein isoforms, in a population of proliferating mononucleated myoblasts with an elongated spindle-shaped morphology without fusion. This may indicate the co-existence of early differentiated but non-fused myoblasts within the population of proliferating myoblasts in adult regenerative muscle. In addition, a small number of the proliferating mononucleated myoblasts expressed myogenin, confirming their differentiated state.

The essential roles of sarcomeric proteins have been highlighted by identification of mutations in their genes associated with various diseases. This includes mutations in genes encoding β-TM and γ-TM isoforms (*TPM2* and *TPM3*) in association with congenital myopathies with a range of clinical and morphological phenotypes [12,13,35-42]. In addition, mutations in *TPM2, TNNI2, MYH3*, *MYH8* and *MYBPC1* have recently been identified to cause the DA syndromes, characterized by congenital contractures [12,15]. The sequential onset of distinct sarcomeric protein isoforms within a family has not been well-characterized in human, except for the *MYH3* and *MYH8,* which are known to be expressed during fetal development and also during muscle regeneration [43,44]. The impact of embryonic and fetal MyHC isoforms for normal fetal development was supported by the identification of *MYH3* and *MYH8* mutations [16,17,45][18,46]. It was suggested that they cause a developmental myopathy resulting in reduced fetal movement and joint contractures [16,17]. Our results here demonstrated the predominant expression of β-TM, cardiac alpha actin, slow TnI and slow MyBPC isoforms in proliferating human mononucleated myoblasts and myotubes during myogenesis *in vitro*. This points to a possible role for these protein isoforms in the early stages of myofibrillogenesis. Mutations in such proteins may affect muscle function during early development either through haploinsufficiency with insufficient dosage of a functional protein or a dominant negative effect of the mutated allele by functional or structural alterations.

## Conclusion

In conclusion, we here confirm that cell cultures of human skeletal muscle are an appropriate tool to study developmental stages of myofibrillogenesis. We show that many proteins involved in muscle diseases are readily detected in the stages of skeletal muscle cell differentiation that can be reached *in vitro.* The early and uniform expression of these proteins suggests their impact on the developmental mechanisms involved in the initial stages of myofibril assembly, differentiation and formation of muscle. This indicates that myoblasts isolated from patients with a mutation in one of the investigated genes may be an invaluable tool to analyze the effects of these mutations on sarcomere assembly and disassembly or myofibril turnover. It would provide us new insights into development of muscle to indicate whether these diseases are disorders of myofibrillogenesis and muscle development.

## Competing interests

The authors declare that they have no competing interests.

## Authors’ contributions

S A-H performed the experiments, assisted in analyzing data and assisted in writing the mauscript; PFMV assisted in analysing data, writing and editing the manuscript; HT performed the study design, analyzed data and wrote and editing the manuscript. Principal investigator and corresponding author. All authors read and approved the final manuscript.

## Pre-publication history

The pre-publication history for this paper can be accessed here:

http://www.biomedcentral.com/1471-2474/13/262/prepub

## Supplementary Material

Additional file 1 **Figure S1.** Sequence analysis of MRF transcripts, TM isoforms, α-skeletal and α-cardiac actin, desmin and titin in both proliferating mononucleated myoblasts and cells after 6 days of differentiation. (A) Sequence chromatograms of part of cDNA of MRFs (*MYOD1*, *MYF5* and *MYOG*) genes. (B) Sequence chromatograms of part of cDNA of different TM isoforms including *TPM1*, *TPM2* and *TPM3*. (C) Sequence chromatograms of part of cDNA of α-skeletal and (*ACTA1*) α-cardiac (*ACTC1*) actin. (D) Sequence chromatograms of part of desmin (*DES*) and titin (*TTN*) genes. The accession number and position of each gene is indicated. Click here for file

Additional file 2 **Figure S2.** Sequence analysis of TnT, TnI and MyBPC isoforms in proliferating mononucleated myoblasts and cells after 6 days of differentiation. (A) Sequence chromatograms of part of cDNA of slow skeletal (*TNNT1*), cardiac (*TNNT2*) and fast skeletal (*TNNT3*) muscle troponin T. (B) Sequence chromatograms of part of cDNA of slow, fast and cardiac troponin I (*TNNI1*, *TNNI2* and *TNNI3*). (C) Sequence chromatograms of part of cDNA of slow and fast skeletal muscle MyBPC (*MYBPC1* and *MYBPC2*) and cardiac-specific (*MYBPC3*) isoforms. The accession number and position of each gene is indicated. Click here for file

Additional file 3 **Figure S3.** Sequence analysis of different MyHC isoforms in proliferating mononucleated myoblasts and cells after 6 days of differentiation. Sequence chromatograms of a specific region of cDNA of different MyHC isoforms including *MYH1, MYH2, MYH3, MYH4, MYH7* and *MYH8.* The accession number and position of each isoform is indicated. Click here for file

Additional file 4 **Figure S4.** Immunofluorescence micrographs of myoblasts and cells after 6 days of differentiation. (Emphasis>/Emphasis>) Proliferating myoblasts stained with antibodies against desmin, (B) embryonic MyHC, (C) neonatal MyHC and (D) fast and slow MyHC, (E) alpha-skeletal actin, (F) all tropomyosin isoforms, (G) beta-tropomyosin isoform, (H) slow troponin I and (I) cardiac troponin I. Cells after 6 days of differentiation stained with antibody against (J) desmin, (K) embryonic MyHC, (L) neonatal MyHC and (M) fast and slow MyHC, (N) alpha-skeletal actin and (O) cardiac troponin I. These staining patterns confirm the data obtained by immunocytochemistry. Nuclei were stained with DAPI (blue). The bar represents 10 μm. Click here for file
