# Supplementary figures and images for: Expression profiles of muscle disease-associated genes and their isoforms during differentiation of cultured human skeletal muscle cells
Source: BMC Musculoskelet Disord. 2012 Dec 29;13:262. doi: 10.1186/1471-2474-13-262 (PMC3549291; doi:10.1186/1471-2474-13-262)

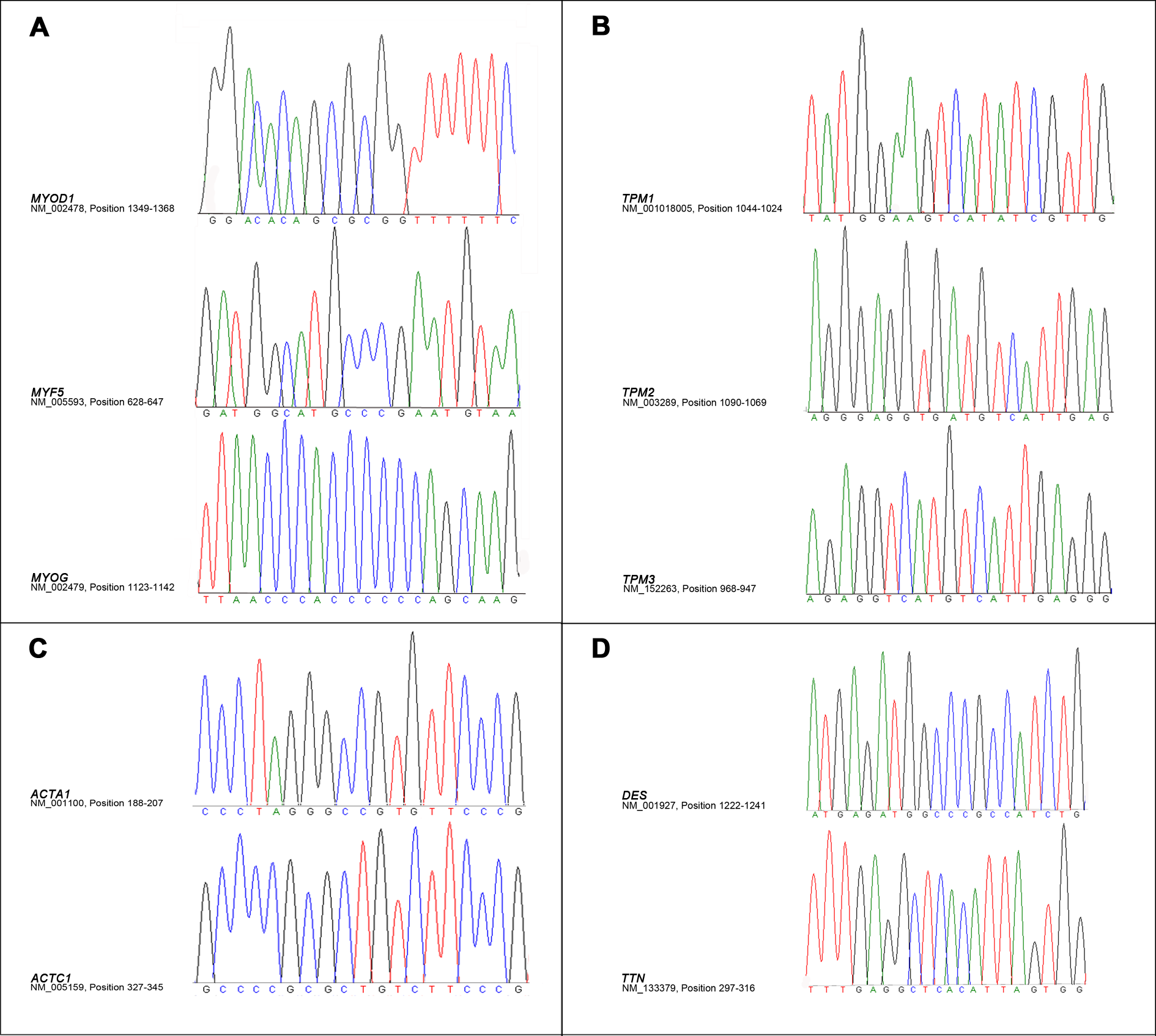

Supplement: Additional file 1 — Figure S1. Sequence analysis of MRF transcripts, TM isoforms, α-skeletal and α-cardiac actin, desmin and titin in both proliferating mononucleated myoblasts and cells after 6 days of differentiation. (A) Sequence chromatograms of part of cDNA of MRFs (MYOD1, MYF5 and MYOG) genes. (B) Sequence chromatograms of part of cDNA of different TM isoforms including TPM1, TPM2 and TPM3. (C) Sequence chromatograms of part of cDNA of α-skeletal and (ACTA1) α-cardiac (ACTC1) actin. (D) Sequence chromatograms of part of desmin (DES) and titin (TTN) genes. The accession number and position of each gene is indicated. [file 1471-2474-13-262-S1.tiff]

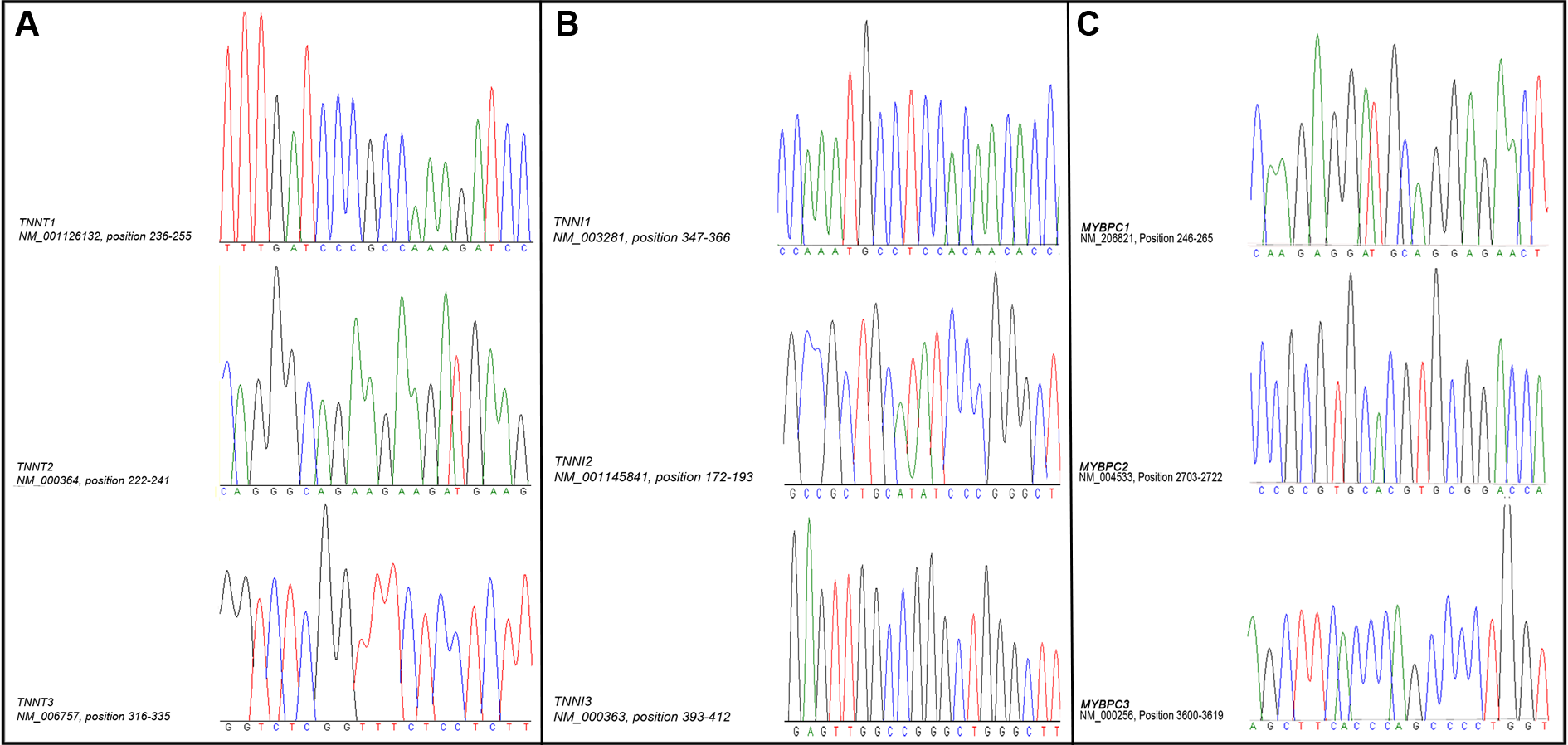

Supplement: Additional file 2 — Figure S2. Sequence analysis of TnT, TnI and MyBPC isoforms in proliferating mononucleated myoblasts and cells after 6 days of differentiation. (A) Sequence chromatograms of part of cDNA of slow skeletal (TNNT1), cardiac (TNNT2) and fast skeletal (TNNT3) muscle troponin T. (B) Sequence chromatograms of part of cDNA of slow, fast and cardiac troponin I (TNNI1, TNNI2 and TNNI3). (C) Sequence chromatograms of part of cDNA of slow and fast skeletal muscle MyBPC (MYBPC1 and MYBPC2) and cardiac-specific (MYBPC3) isoforms. The accession number and position of each gene is indicated. [file 1471-2474-13-262-S2.tiff]

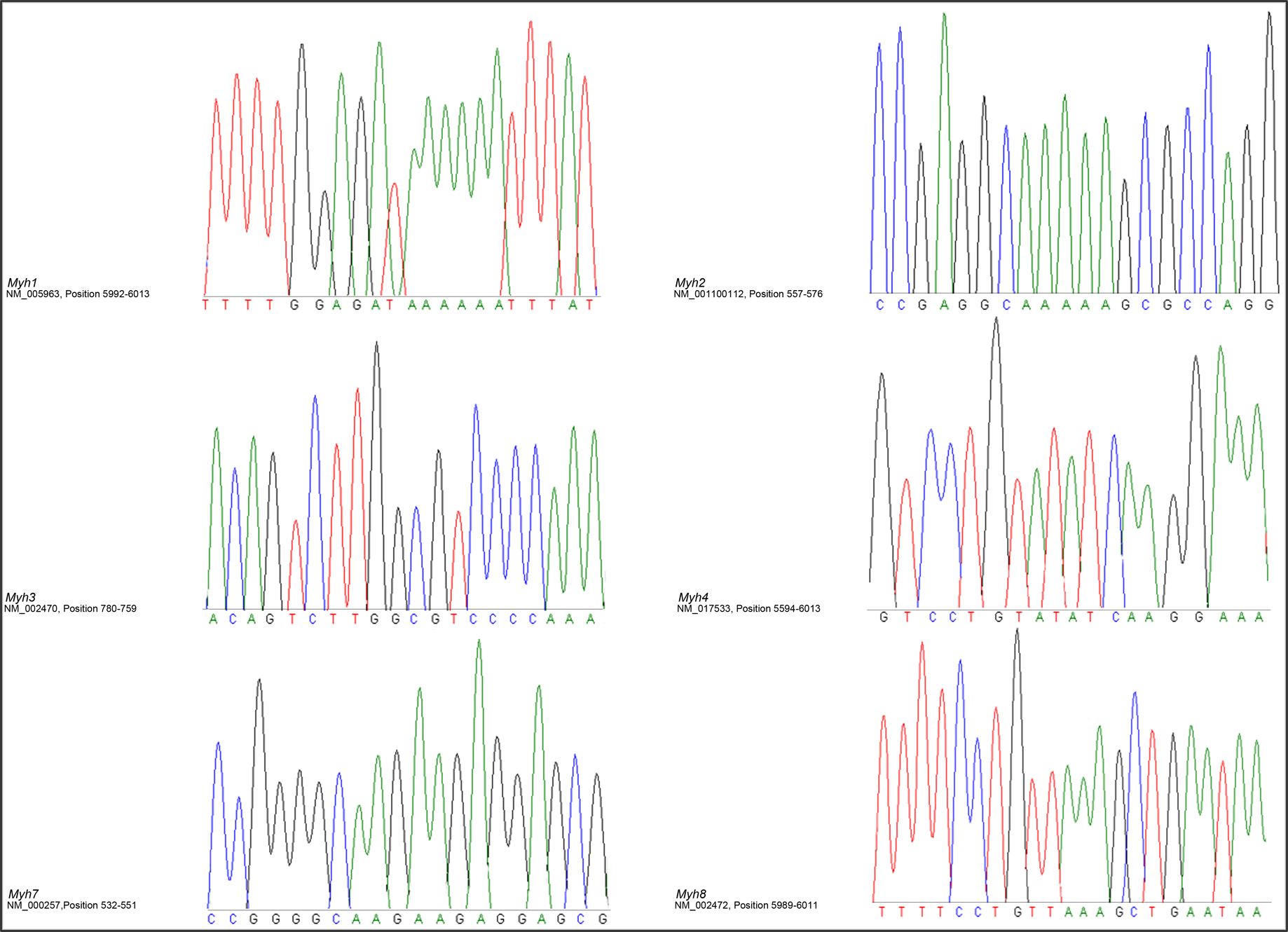

Supplement: Additional file 3 — Figure S3. Sequence analysis of different MyHC isoforms in proliferating mononucleated myoblasts and cells after 6 days of differentiation. Sequence chromatograms of a specific region of cDNA of different MyHC isoforms including MYH1, MYH2, MYH3, MYH4, MYH7 and MYH8. The accession number and position of each isoform is indicated. [file 1471-2474-13-262-S3.tiff]

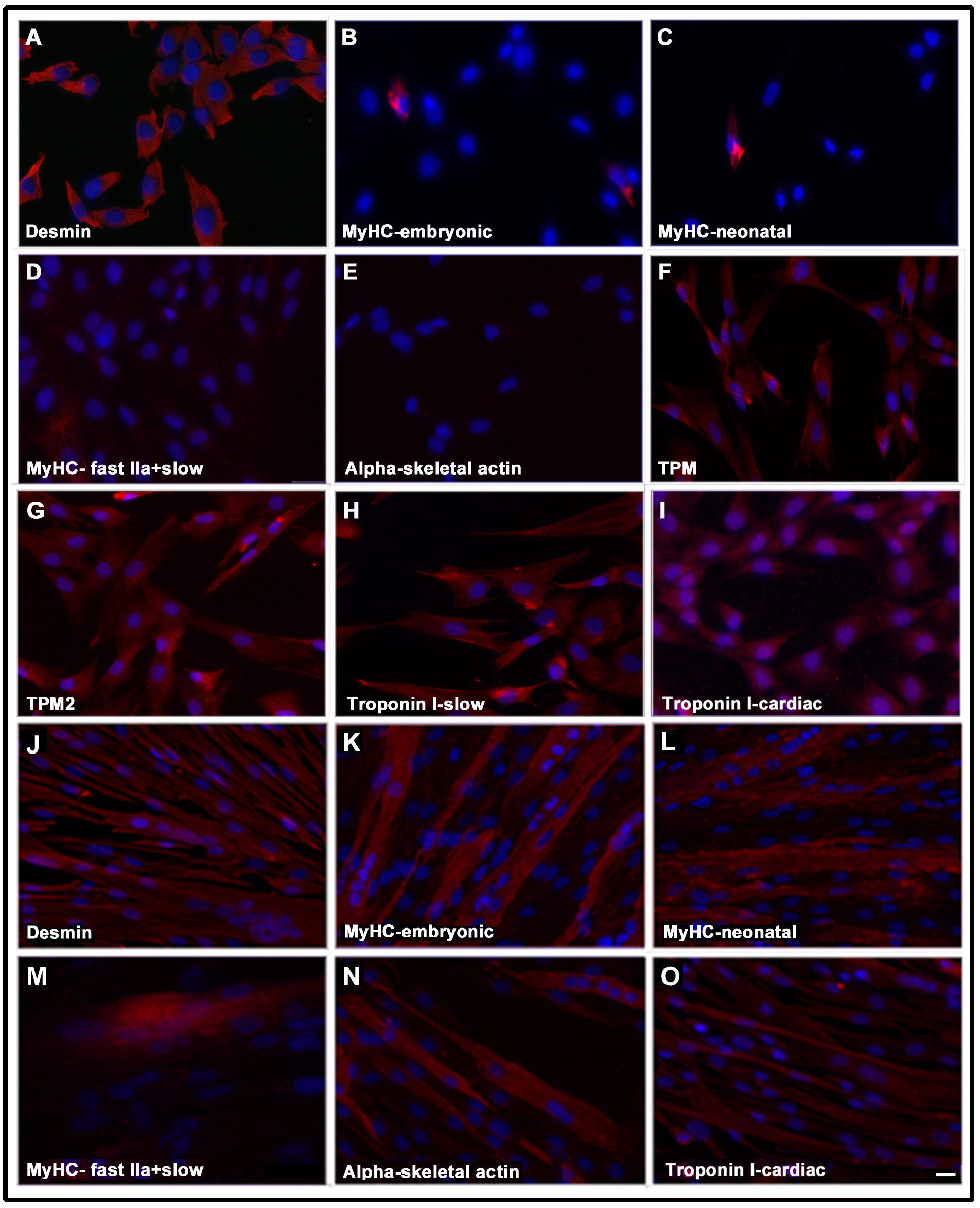

Supplement: Additional file 4 — Figure S4. Immunofluorescence micrographs of myoblasts and cells after 6 days of differentiation. (Emphasis>/Emphasis>) Proliferating myoblasts stained with antibodies against desmin, (B) embryonic MyHC, (C) neonatal MyHC and (D) fast and slow MyHC, (E) alpha-skeletal actin, (F) all tropomyosin isoforms, (G) beta-tropomyosin isoform, (H) slow troponin I and (I) cardiac troponin I. Cells after 6 days of differentiation stained with antibody against (J) desmin, (K) embryonic MyHC, (L) neonatal MyHC and (M) fast and slow MyHC, (N) alpha-skeletal actin and (O) cardiac troponin I. These staining patterns confirm the data obtained by immunocytochemistry. Nuclei were stained with DAPI (blue). The bar represents 10 μm. [file 1471-2474-13-262-S4.tiff]
